# Cerebral blood flow dynamics: Is there more to the story at exercise onset?

**DOI:** 10.14814/phy2.15735

**Published:** 2023-06-07

**Authors:** John Ashley, Joe Shelley, Jiwon Song, Jongjoo Sun, Rebecca D. Larson, Daniel J. Larson, Ari Berkowitz, Andriy Yabluchanskiy, J. Mikhail Kellawan

**Affiliations:** ^1^ Institute for Exercise and Environmental Medicine Texas Health Presbyterian Hospital Dallas Texas USA; ^2^ Department of Neurology and Neurotherapeutics University of Texas Southwestern Medical Center Dallas Texas USA; ^3^ Department of Health and Exercise Science, Human Circulation Research Laboratory University of Oklahoma Norman Oklahoma USA; ^4^ Department of Health and Exercise Science, Body Composition and Physical Performance Research Laboratory University of Oklahoma Norman Oklahoma USA; ^5^ Department of Health and Exercise Science, Sport, Health, and Exercise Data Analytics Laboratory University of Oklahoma Norman Oklahoma USA; ^6^ Department of Biology and Cellular and Behavioral Neurobiology Graduate Program University of Oklahoma Norman Oklahoma USA; ^7^ Oklahoma Center for Geroscience and Healthy Brain Aging, Department of Neurosurgery University of Oklahoma Health Sciences Center Oklahoma City Oklahoma USA

## Abstract

A monoexponential model characterizing cerebral blood velocity dynamics at the onset of exercise may mask dynamic responses by the cerebrovasculature countering large fluctuations of middle cerebral artery blood velocity (MCAv) and cerebral perfusion pressure (CPP) oscillations. Therefore, the purpose of this study was to determine whether the use of a monoexponential model attributes initial fluctuations of MCAv at the start of exercise as a time delay (TD). Twenty‐three adults (10 women, 23.9 ± 3.3 yrs; 23.7 ± 2.4 kg/m^2^) completed 2 min of rest followed by 3 mins of recumbent cycling at 50 W. MCAv, CPP, and Cerebrovascular Conductance index (CVCi), calculated as CVCi = MCAv/MAP × 100 mmHg, were collected, a lowpass filter (0.2 Hz) was applied, and averaged into 3‐second bins. MCAv data were then fit to a monoexponential model [ΔMCAv(*t*) = Amp(1 – e^−(*t*−TD)/τ^)]. TD, tau (τ), and mean response time (MRT = TD + τ) were obtained from the model. Subjects exhibited a TD of 20.2 ± 18.1 s. TD was directly correlated with MCAv nadir (MCAv_N_), *r* = −0.560, *p* = 0.007, which occurred at similar times (16.5 ± 15.3 vs. 20.2 ± 18.1 s, *p* = 0.967). Regressions indicated CPP as the strongest predictor of MCAv_N_ ($R_{a}^{2}$ = 0.36). Fluctuations in MCAv were masked using a monoexponential model. To adequately understand cerebrovascular mechanisms during the transition from rest to exercise, CPP and CVCi must also be analyzed. A concurrent drop in cerebral perfusion pressure and middle cerebral artery blood velocity at the start of exercise forces the cerebrovasculature to respond to maintain cerebral blood flow. The use of a monoexponential model characterizes this initial phase as a time delay and masks this large important response.

## 1 INTRODUCTION

Analyzing the kinetics of physiological responses to exercise has allowed for greater understanding and new insights related to oxygen consumption (VO_2_), oxygenation, and peripheral blood flow control in dynamic settings (Barbosa et al., 2011; Poole & Jones, 2013; Tschakovsky et al., 2006). Recently, a monoexponential model has been applied to analyze middle cerebral artery blood velocity (MCAv) data from a rest‐to‐exercise transition using various large muscle mass exercise protocols (Billinger et al., 2017; Weston et al., 2022). Since then, several studies published using this analysis technique comparing various populations suggest differences exist with time delay (TD) in health and disease (Kaufman et al., 2019; Ward et al., 2018, 2022; Weston et al., 2022; Witte et al., 2019). These data are important to gain a greater understanding of the dynamic adjustment of MCAv. However, several of the data put forth warrant more investigation to better understand what these differences mean and how to properly interpret these results.

When MCAv data from a rest‐to‐exercise transition are fit with a monoexponential model, the TD for young healthy adults is reported to be 30–50 s from the start of exercise to the model‐detected increase in MCAv (Billinger et al., 2017; Ward et al., 2018; Weston et al., 2022). It is unlikely the cerebrovasculature is less responsive (TD ~ 10 s) than other systems (Craig et al., 2021; MacDonald et al., 1998; Saunders et al., 2005; Tschakovsky et al., 2006) given the tight flow‐metabolism coupling within the brain (Sheth et al., 2004; Willie et al., 2014). A more reasonable assumption is the oscillations of MCAv at the start of exercise are characterized as a TD when using a monoexponential model. As first pointed out by Weston et al. (2022), this initial fluctuation lasts around 25 s. They speculated this was a result of changes pressure and end‐tidal CO_2_ (EtCO_2_). Both CO_2_ and pressure are strong vasoactive stimuli within the cerebrovasculature (Caldwell et al., 2021; Poole & Jones, 2013; Willie et al., 2014). However, given the exercise‐induced hypotension at the start of large muscle mass exercise, (Craig et al., 2021; Ogoh et al., 2022; Wieling et al., 1996), and the synchronization between pressure and MCAv (Favre & Serrador, 2019; Kay & Rickards, 2016; Labrecque et al., 2019), we believe pressure is the primary contributor to the initial MCAv fluctuations, and therefore, the extended TD being reported.

Therefore, the purpose of this study was to analyze the initial oscillations observed with MCAv recordings at the start of cycling exercise and determine whether these oscillations are a result of a transient exercise‐induced hypotension. We hypothesized that at the beginning of exercise, MCAv will show a transient but significant drop prior to a monoexponential increase. We also hypothesized that this drop is attributable to a concurrent drop in cerebral perfusion pressure (CPP).

## 2 METHODS

### 2.1 Participants

Thirty‐two young (18–30 years; 16 Females, 16 Males), healthy (BMI < 30 kg/m^2^) subjects were recruited for the current study. Graded‐exercise testing data were previously published (Ashley et al., 2020). Two subjects (1 female and 1 male) were removed due to equipment malfunction, and 7 subjects (5 females and 2 Males) were removed due to non‐exponential increase, or model failure (see *MCAv Kinetic Analysis*). Therefore, 23 subjects (10 females and 13 males, Table 1) were analyzed in the present study. All subjects reported being free of cardiovascular, metabolic, or respiratory diseases, physical ailments, and were not considered sedentary (>600 MET‐min/wk) as determined by a medical history questionnaire and the International Physical Activity Questionnaire (iPAQ) long form. Females with a normal menstruating cycle were studied within the early follicular phase (1–7 days) of their menstrual cycle or during the placebo phase of their oral contraceptives to minimize gonadal hormone influences on vasculature (Hashimoto et al., 1995). All study procedures were approved by the Institutional Review Board at the University of Oklahoma Health Sciences Center (IRB# 10121) and conformed to the standards set by the Declaration of Helsinki with the exception of registration in a database.

**TABLE 1:** Subject demographic data.

Parameter	Total	Slow	Fast
Subjects (*N*)	23 (*F* = 10)	15 (*F* = 7)	8 (*F* = 3)
Age (yrs)	23.9 (3.3)	24.0 (3.4)	23.9 (3.3)
Height (cm)	175.1 (7.9)	174.6 (9.4)	176.0 (4.1)
Weight (kg)	72.8 (10.4)	72.3 (10.9)	73.8 (10.0)
BMI (kg/m^2^)	23.7 (2.4)	23.6 (2.6)	23.8 (2.3)
Triglycerides (mg/dL)	74.4 (22.0)	69.1 (12.1)	84.2 (32.6)
HDL (mg/dL)	56.8 (12.4)	55.0 (10.5)	60.2 (15.5)
LDL (mg/dL)	63.9 (19.0)	60.4 (21.3)	71.1 (11.3)
Glucose (mg/dL)	92.5 (7.6)	92.0 (8.6)	93.3 (5.6)
iPAQ (MET‐min/wk)	4563 (3333)	5549 (3442)	2714 (2287)*
VO_2PEAK_ (mL/kg/min)	34.2 (8.7)	35.6 (9.5)	31.6 (6.6)
Baseline			
HR (bpm)	79.8 (14.5)	78.8 (15.0)	81.6 (14.3)
MAP (mmHg)	92.2 (16.5)	88.7 (11.8)	98.9 (22.4)
MCAv (cm/s)	65.4 (14.0)	67.6 (13.2)	61.2 (15.4)

*Note*: Values mean (SD) unless stated otherwise. “Total” represents the total sample; “slow” individuals with TD > 1.5 s; “fast” individuals with TD ≤ 1.5 s. Subjects are presented as sample size with women in parentheses. Baseline data are the averaged last 30 s prior to exercise start. *Denotes significantly different from slow, *p* < 0.05.

### 2.2 Protocol

All participants completed two laboratory visits. For both visits, participants were asked to arrive ≥8 h fasted, ≥12 h without caffeine, and ≥24 h without exercise, alcohol, or the use of supplements or nonsteroidal anti‐inflammatory drugs. On the first visit, participants provided written informed consent and completed medical history and iPAQ questionnaires. Following consent, participants' height (Novel Products, Inc.), weight (BWB‐800A, Tanita), resting blood pressure, and a venous blood sample were collected. Resting blood pressure was measured following ≥5 mins of supine rest using an automated upper arm cuff (HEM‐705, Omron). Blood was analyzed (CardioCheck PA, Poymer Technology Systems Inc.) for fasting glucose <100 mg/dL, triglycerides <150 mg/dL, HDL ≥40 mg/dL in males or ≥50 mg/dL in females for inclusion. Participants were then familiarized with the study equipment and procedures, and a quality transcranial doppler (TCD) signal of the MCA was confirmed. On the second visit, participants arrived at the laboratory, as previously requested, were fitted with the study equipment and sat quietly on a recumbent cycle ergometer (Lode Corival cpet). After at ≥2 min of quiet rest (Baseline), subjects immediately began peddling at 50 W between 60 and 80 RPM 3 min.

### 2.3 Study measurements

Middle cerebral artery blood velocity was continuously measured using bilateral TCD ultrasound probes (2 MHz pulsed‐wave Robotic TCD probe, Neurovision, Multigon Industries). Both the left and right MCA were insonated according to previously published guidelines (Aaslid et al., 1982; Willie et al., 2011). Infrared finger photoplethysmography (Human NIBP Nano System, ADInstruments) was used for continuous measurement of mean arterial pressure (MAP) with in‐software calculations for estimates of cardiac output (Q), stroke volume (SV), and total peripheral resistance (TPR). Heart rate (HR) was continuously recorded using a wireless ECG vest (EQ02 + *SEM*, Equivital). Pulmonary gases and flow were continuously analyzed (Gemini End‐Tidal O_2_ and CO_2_ Analyzer, CWE Inc.; MLT3813H Pneumotach, ADInstrumetns; FE141 Spirometer, ADInstruments) for breath‐by‐breath analysis of VO_2_ and EtCO_2_. All data were acquired via PowerLab (PowerLab/16SP ML 880, ADInstruments) at 20 kHz recorded using LabChart software (ADInstruments).

### 2.4 Data analysis

**FIGURE 1:**
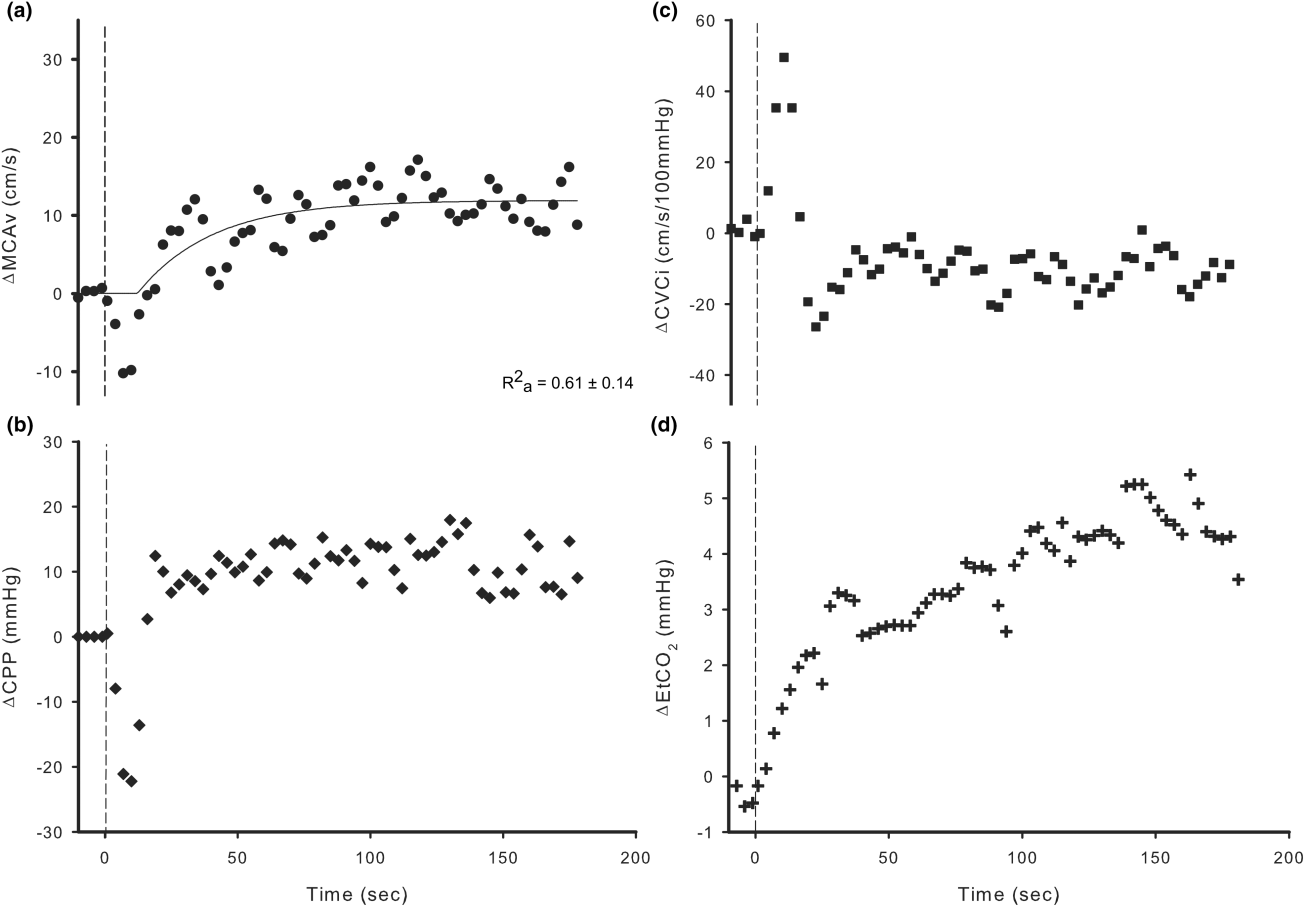
Change from rest (set to 0) to immediate 50 W cycling exercise for middle cerebral artery blood velocity (MCAv; a), cerebral perfusion pressure (CPP; b), cerebrovascular conductance index (CVCi; c), and end‐tidal CO_2_ (EtCO_2_; d). Exercise start is indicated by the vertical dashed lines. All data sets presented are averaged 3‐s bins and from representative subjects. Solid line on graph A represents best fit using a monoexponential model. Adjusted *R* ^2^ ($R_{a}^{2}$) = 0.61 ± 0.14. Standard Error of Regression (S) = 3.74 ± 0.91.

Data were exported in raw format (20 kHz), and a lowpass filter (set at 0.2 Hz) was applied (Ferreira et al., 2006). These data were then averaged into 3‐s time bins for comparative analysis (Billinger et al., 2017). Both left and right MCA were insonated and averaged together to form a single response. For twelve subjects, only one MCA was insonated, that side was used for data analysis. The last 30 s prior to the start of exercise was averaged for baseline. CPP was calculated as [CPP = MAP – (0.7355 × height in cm from heart to TCD probe)] to account for the hydrostatic column (Deegan et al., 2010; Des, 2012; Favre et al., 2020). Cerebral vascular conductance index (CVCi) was calculated as (CVCi = MCAv/CPP × 100 mmHg). Several points of interest were identified and used for analysis. Immediately at the start of exercise, both MCAv and CPP dropped to a nadir (MCAv_N_, CPP_N_), and CVCi increased towards a maximum (CVCi_M_, see Figure 1). To assess the ability of cerebral autoregulation to maintain CBF during the rapid changes in perfusion pressure at the onset of exercise, autoregulatory compensation index (ARCi) was calculated as

$$\text{ARCi}=\left[1–\left({\text{MCAv}}_{\mathrm{EN}}/{\text{MCAv}}_{N}\right)\right]\times 100.$$

where estimated MCAv nadir (MCAv_EN_) is the value MCAv would have reached if there were no cerebrovascular response and CVCi had maintained baseline levels. This was calculated as MCAv_EN_ = (CVCi_BL_ × CPP_@MCAvN_)/100. Effectively, if CVCi had not changed from baseline (CVCi_BL_), MCAv_EN_ is the value MCAv *would* have reached when CPP changed at the same time point MCAv_N_ occurred (CPP_@MCAvN_). This calculation was used to determine the buffering capacity of autoregulatory mechanisms to prevent a further decrease in MCAv.

### 2.5 MCAv kinetic analysis

The rest‐to‐exercise MCAv transition data were fit with a monoexponential model using SigmaPlot 12.5 (Systat Software, Inc.) as previous research has shown (Billinger et al., 2017; Witte et al., 2019). The model used was the following:

$$\text{ΔMCAv}\left(t\right)=\mathrm{BL}+\mathrm{Amp}\left(1-e^{-\left(t–\mathrm{TD}\right)/\tau }\right)$$

where ΔMCAv(*t*) is the change in MCAv from baseline to any given timepoint, BL is the baseline prior to exercise start (data are baseline corrected; therefore, baseline is set to 0), Amp is the change from baseline to the steady‐state MCAv, TD is the time prior to an increase in MCAv, and tau (τ) is the time constant from the response to reach 63% of steady state. Mean response time (MRT) was calculated by adding TD and tau. This gave a value representing the total time from the start of exercise, until MCAv reached 63% of the steady‐state value (Phillips et al., 1995). Model fit was assessed through visual inspection of the model, $R_{a}^{2}$ value, and equivalence of variance in residuals, tested using SigmaPlot's Constant Variance Test which utilizes a Spearman rank correlation between the absolute values of the residuals and the observed value of the dependent variable. If it was found that a given data set had unequal variance in residuals, it was considered a “model failure” and removed from analysis.

### 2.6 Statistical analysis

The cerebral hemodynamic responses (MCAv_N_, CPP_N_, and CVCi_M_) were correlated with model characteristics (TD, tau, and MRT) to determine their contributions to changes in model results. Two‐tailed dependent samples *t*‐tests were used to determine whether MCAv_N_ occurred at similar times to CPP_N_, CVCI_M_, and TD. If any of the *t*‐tests revealed no significance (*p* > 0.05), a two one‐sided test (TOST) was completed to determine whether the means were equivalent (Lakens, 2017). Using a sample size of *N* = 23, α = 0.05, and power of 80%, the smallest effect size of interest was calculated as *d* = 0.611, which was used in the TOST procedure. A more in‐depth description of this type of analysis and its results can be found elsewhere (Lakens, 2017). Briefly, TOST calculates two *p* values based on two one‐sided *t*‐tests. This test determines whether the difference between the two samples is greater than an equivalence boundary based on the effect size. If both *t*‐tests are significant (i.e., both *p* values are below 0.05), the data are considered equivalent. Only the largest *p* value of the two tests is reported, as follows:

$$t\left(\mathrm{df}\right)=\text{test statistic},p\;\text{value}$$

This means, if the *p* value reported is significant, the data are considered equivalent. There are scenarios where significance test and TOST results are at odds. We will follow the recommendations as outlined here (Lakens, 2017).

The following regression was calculated to describe MCAv_N_:

$$\begin{array}{c} {\text{ΔMCAv}}_{N}={\text{ΔCPP}}_{@\text{MCAvN}}+{\text{ΔCVCi}}_{@\text{MCAvN}}+{\mathrm{\Delta Q}}_{@\text{MCAvN}}\\ \;+{\text{ΔTPR}}_{@\text{MCAvN}}+{\text{ΔEtCO}}_{2@\text{MCAvN}} \end{array}$$

where each variable is the change from baseline to the timepoint MCAv_N_ occurred. Each variable was chosen due to previous research showing a strong relationship with CBF (Markwalder et al., 1984; Ogoh et al., 2005). Due to the results of the regression, a follow‐up regression with CPP_@MCAvN_ only was completed.

Additionally, retrospective analysis of the data revealed a large distribution of subjects experienced a rapid MCAv response (Figure S1) as the monoexponential model detected the first data point used in analysis (at 1.5 s) as the increase. Therefore, these subjects were separated into “fast” (TD ≤ 1.5 s) and “slow” (TD > 1.5 s) groups. Model characteristics were compared between the groups using a Welch's *t*‐test. TOST was completed for any non‐significant results (*p* > 0.05). With two samples, *N* = 8 and *N* = 15 (fast and slow groups, respectively), α = 0.05 and Power set to 80%, the smallest effect size of interest was calculated as *d* = 1.32, which was used in the TOST procedure.

Lastly, although the purpose of this study was not to determine whether sex differences exist in cerebral hemodynamics, the nearly‐even split between males (*N* = 13) and females (*N* = 10) allowed us the opportunity to explore this. A TOST equivalence test was used to determine whether full statistical analysis was needed to determine differences between the sexes (O'Brien & Kimmerly, 2022). Considering the sample size and given a power of 80% with an α of 0.05, an effect size of *d* = 1.26 would be needed to determine differences between the sexes.

Statistical analyses were completed using R (R Core Team, 2022) software. All regressions (including the monoexponential model) are reported with adjusted R^2^ ($R_{a}^{2}$) to eliminate as much bias as possible and to be used for comparisons across various regression types (Akossou & Palm, 2013), and standard error of the regression (S). All TOST results are reported as *t*(df) = test statistic, *p*, where the test statistic and *p* value reported are the largest of the two *t*‐tests (Lakens, 2017). All data are reported as means ± SD with significance set at *p* < 0.05.

## 3 RESULTS

### 3.1 Study participants

Characteristics of 23 subjects (10 females) are presented in Table 1. The work rate (50 W) equated to 28.1 ± 6.9% of their max (187.0 ± 39.4 W) output or 37.5 ± 8.3% VO_2Peak_ which is considered light intensity exercise (American College of Sports Medicine et al., 2021).

### 3.2 Model characteristics

The MCAv response to 50 W cycling was fit well by a monoexponential model ($R_{a}^{2}$ = 0.61 ± 0.14, S = 3.74 ± 0.91, Figure 1a). The MCAv kinetic parameters are displayed in Table 2.

**TABLE 2:** Model characteristics.

Model variables	Mean (SD)	SE (min, max)
$R_{a}^{2}$	0.61 (0.14)	3.74 (1.55, 5.38)*
Amp (Δcm/s)	12.8 (5.8)	1.60 (0.88, 4.33)
TD (s)	20.2 (18.1)	6.52 (0.81, 12.81)
tau (s)	29.4 (23.0)	14.62 (1.73, 36.34)
MRT (s)	49.4 (23.6)	

*Note*: Parameters from monoexponential model fit of change of middle cerebral artery blood velocity (ΔMCAv) from rest to exercise. Adjusted *R* ^2^ ($R_{a}^{2}$), amplitude (Amp), time delay (TD), mean response time (MRT); Data are represented as mean (SD). *$R_{a}^{2}$ error is standard error of regression (S).

Points of interest on the cerebrovascular responses were MCAv_N_ (−4.0 ± 5.5 Δcm/s), CPP_N_ (−13.5 ± 10.4 ΔmmHg), and CVCi_M_ (27.3 ± 24.6 Δcm/s/100 mmHg), as can be seen in Figure 1. The only correlations that were significant were between MCAv_N_ and TD (*r* = −0.560, *p* = 0.007) and tau (*r* = 0.434, *p* = 0.039), Table 3. The time MCAv_N_ occurred and the recorded TD were not different (16.5 ± 15.3 vs. 20.2 ± 18.1 s, *p* = 0.967, time of MCAv_N_ vs. TD) and were equivalent (*t*(22) = 2.053, *p* = 0.026). The time MCAv_N_ and the time CPP_N_ occurred tended to be different (16.5 ± 15.3 vs. 10.5 ± 4.5 s, *p* = 0.07, *d* = 0.55) and they were not equivalent (*t*(22) = −0.877, *p* = 0.195) suggesting CPP_N_ may have occurred earlier than MCAv_N_. CVCi_M_ occurred after MCAv_N_ (46.9 ± 57.0 vs. 16.5 ± 15.3 s, *p* = 0.02, *d* = 0.73, time of CVCi_M_ vs. time of MCAv_N_, respectively).

**TABLE 3:** Correlation between model characteristics and cerebral hemodynamics.

	TD (s)	tau (s)	MRT (s)
MCAv_N_ (cm/s)	−0.560*	0.434*	−0.021
CPP_N_ (mmHg)	−0.306	−0.071	−0.310
CVCi_M_ (cm/s/100 mmHg)	0.153	−0.148	−0.023

*Note*: Data are correlation coefficients for monoexponential model parameters (time delay, TD, tau, and mean response time, MRT) and cerebrovascular parameters (CPP_N_, cerebral perfusion pressure nadir; CVCi_M_, cerebrovascular conductance index maximum; MCAv_N_, middle cerebral artery blood velocity nadir). *Denotes significance *p* < 0.05.

### 3.3 CPP is the primary predictor of MCAv_N_

To explain MCAv_N_, a regression of parameters occurring at MCAv_N_ was completed (See methods for more detail). The results of this regression are presented in Table 4.

**TABLE 4:** Results from multiple linear regression of MCAv nadir.

	ΔCPP_@MCAvN_	ΔCVCi_@MCAvN_	ΔQ_@MCAvN_	ΔTPR_@MCAvN_	ΔEtCO_2@MCAvN_
MCAv_N_ ($R_{a}^{2}$ = 0.36, S = 4.97, AIC = 140.44)					
β Coefficient	0.58 (0.16)*	0.16 (0.06)*	0.20 (0.54)	−0.009 (0.03)	0.41 (0.46)

*Note*: Results from multiple linear regression modeling of middle cerebral artery blood velocity (MCAv). Values are reported as β coefficient (SE). MCAv_N,_ the lowest point MCAv achieved during 50 W cycling was regressed with the responses of cerebral perfusion pressure at MCAv_N_. (ΔCPP_@MCAvN_), cerebrovascular conductance index at MCAv_N_ (ΔCVCi_@MCAvN_), cardiac output at MCAv_N_ (ΔQ_@MCAvN_), total peripheral resistance at MCAv_N_ (ΔTPR_@MCAvN_), and End‐tidal CO_2_ at MCAv_N_ (ΔEtCO_2@MCAvN_). Adjusted *R* ^2^ ($R_{a}^{2}$), standard error of regression (S), Akaike information criterion (AIC). *Indicates significance *p* < 0.05.

**FIGURE 2:**
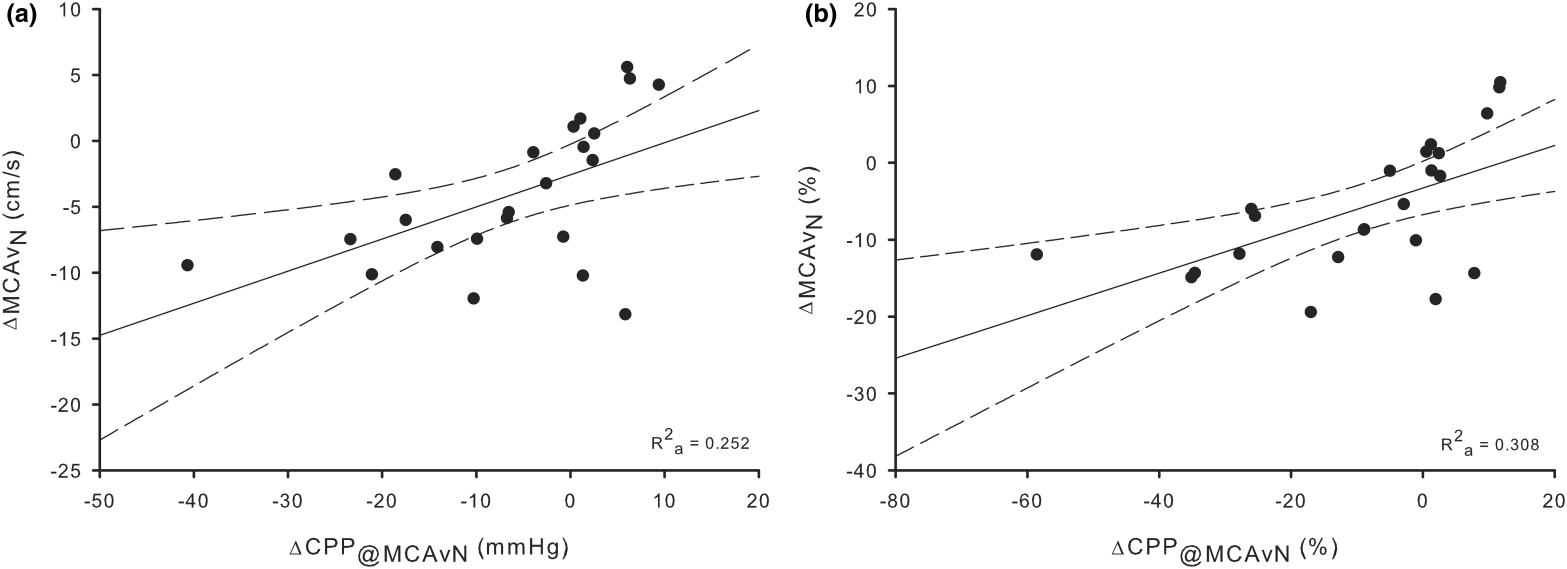
Linear regression between MCAv nadir (MCAv_N_) and CPP at MCAv_N_ (CPP_@MCAvN_) absolute change (a) and percent change (b) from baseline. Solid line represents line of best fit. Dashed lines represent 95% confidence intervals. Adjusted *R* ^2^ ($R_{a}^{2}$) = 0.25, *p* = 0.008, S = 4.74, AIC = 140.7 (a), $R_{a}^{2}$ = 0.36, *p* < 0.001, S = 7.09, AIC = 140.7 (b).

This model indicates only CPP and CVCi were significant predictors of MCAv_N_ at *p* < 0.05. However, due to the way CVCi is calculated (CVCi = MCAv/CPP), it will inherently be related to MCAv and presents a mathematical issue for interpretation. Therefore, we completed a regression with CPP_@MCAvN_ as the only predictor (Figure 2a). The overall fit of this model compared to the larger model was similar ($R_{a}^{2}$ = 0.36 vs. 0.25; S = 4.97 vs. 4.74; AIC = 140.4 vs. 140.7, MCAv_N_ regression with full parameters vs. CPP only). Additionally, and mostly for comparative purposes, the same regression was completed using the percentage change values (Figure 2b). The calculated ARCi was 3.5 ± 16.8%.

### 3.4 Characteristics for fast vs. slow responders

**FIGURE 3:**
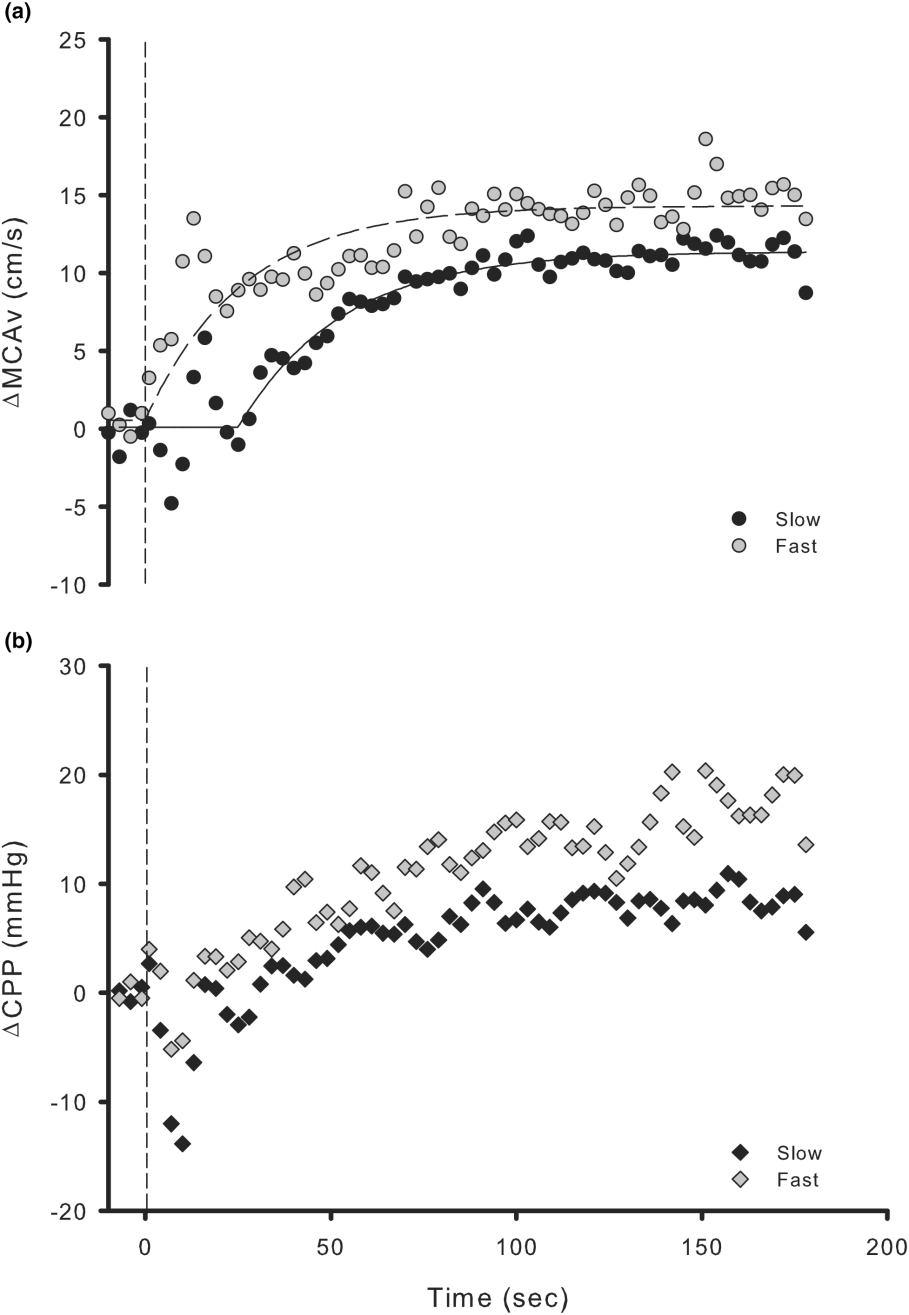
Comparison of middle cerebral artery blood velocity (MCAv, a) and cerebral perfusion pressure (CPP, b) responses from rest to exercise between fast (open, *N* = 8) and slow (filled in, *N* = 15) groups. Dashed line indicates the start of exercise. Displayed data are the averaged data for each group.

As explained, eight subjects (fast) exhibited a rapid MCAv response at the start of exercise (Figure 3a). Demographics comparing the fast and the slow groups are presented in Table 1. These groups had similar distributions of females (46.7 vs. 37.5% females, slow vs. fast); fitness levels were not different (35.6 vs. 31.6 VO_2Peak_ mL/kg/min, *p* = 0.309) and equivalent (*t*(21) = −1.972, *p* = 0.0309); and resting MCAv was not different (67.6 vs. 61.2 cm/s, *p* = 0.311) and equivalent (*t*(21) = −1.976, *p* = 0.0307) between the groups. Only physical activity (5549 vs. 2741 MET‐min/wk, *p* = 0.049) seemed to be different.

### 3.5 Slow responders had greater MCAv fluctuations

The overall response of MCAv to 50 W cycling was not different between the two groups (11.6 ± 6.4 vs. 15.5 ± 4.8 Δcm/s, *p* = 0.143, slow vs. fast) and tended to be equivalent (*t*(21) = 1.493, *p* = 0.0751), whereas the overall response of CPP was greater in the fast group (8.6 ± 8.6 vs. 17.3 ± 8.4 ΔmmHg, *p* = 0.030, slow vs. fast). Model characteristics of both groups are presented in Table 5.

**TABLE 5:** Model characteristics between fast and slow groups.

Parameter	Slow (*N* = 15)	Fast (*N* = 8)	*p*	*d*
$R_{a}^{2}$	0.59 (0.16)	0.66 (0.07)	0.296	0.46
Amp (Δcm/s)	11.7 (6.4)	15.0 (4.0)	0.193	0.59
TD (s)	30.1 (14.6)	1.5 (0.0)*	<0.001	2.41
tau (s)	22.9 (21.5)	41.6 (21.9)	0.061	0.86
MRT (s)	53.1 (24.4)	42.5 (21.9)	0.280	0.49

*Note*: Data are of slow (individuals with a TD >1.5 s) and fast (individuals with a TD ≤1.5 s). Parameters from monoexponential model fit of change of middle cerebral artery blood velocity (ΔMCAv) from rest to exercise. Adjusted *R* ^2^ ($R_{a}^{2}$), Amplitude (Amp), Time delay (TD), mean response time (MRT), and tau are variables gathered from monoexponential model fitting. Data are represented as mean (SD). *Denotes significant difference from slow, *p* < 0.05.

Both groups displayed a good model fit that was not different from each other ($R_{a}^{2}$ = 0.59 ± 0.16 vs. 0.66 ± 0.07, *p* = 0.296, S = 3.71 ± 0.95 vs. 3.80 ± 0.99, *p* = 0.813). As expected, TD was different (30.1 ± 14.3 vs. 1.5 ± 0.0 s, *p* < 0.001, *d* = 2.41) between the groups. Tau tended to be different (22.9 ± 21.5 vs. 41.6 ± 21.9 s, *p* = 0.061, *d* = 0.86, slow vs. fast) and was not equivalent (*t*(21) = 1.043, *p* = 0.155) suggesting tau may have been different between the groups. Lastly, MRT was not different (53.1 ± 24.4 vs. 42.5 ± 21.9 s, *p* = 0.279, *d* = 0.49, slow vs. fast) and was equivalent (*t*(21) = −1.995, *p* = 0.030). Exemplifying the relationship between TD and MCAv_N_, the two groups experienced different MCAv_N_ (−7.3 ± 3.4 vs. 2.0 ± 2.6 Δcm/s, *p* < 0.001, *d* = 2.91, slow vs. fast, Table 6). Although the CPP_N_ between the groups was not different (−8.8 ± 11.2 vs. −16.1 ± 9.3 ΔmmHg, *p* = 0.106, *d* = 0.739), these groups were not equivalent (*t*(21) = 1.326, *p* = 0.099), suggesting the slow group may have experienced a larger CPP_N_. The vascular responses during this time, ARCi and CVCi, were opposite during the MCAv_N_ between the two groups (Table 6) as they were experiencing opposing CPP_@MCAvN_ stimulus (−11.3 ± 11.8 vs. 3.7 ± 3.2 ΔmmHg, *p* < 0.001, *d* = 1.53, slow vs. fast).

**TABLE 6:** Comparison between fast and slow groups.

Parameter	Slow (*N* = 15)	Fast (*N* = 8)	*p*	*d*
ΔMCAv (cm/s)	11.6 (6.4)	15.5 (4.8)	0.143	0.67
ΔCPP (mmHg)	8.6 (8.6)	17.3 (8.4)*	0.030	1.02
ΔCVCi (cm/s/100 mmHg)	3.2 (15.7)	0.1 (6.6)	0.600	0.23
ΔMCAv_N_ (cm/s)	−7.3 (3.4)	2.0 (2.6)*	<0.001	2.91
ΔCPP_N_ (mmHg)	−16.1 (9.3)	−8.7 (11.2)	0.106	0.74
ΔCPP_@MCAvN_ (mmHg)	−11.3 (11.8)	3.7 (3.2)*	<0.001	1.53
ΔCVCi_@MCAvN_ (cm/s/100 mmHg)	11.0 (26.2)	−1.4 (1.8)	0.099	0.60
AACi (%)	6.4 (20.4)	−2.0 (1.9)	0.266	0.50

*Note*: Data are of slow (individuals with a TD >1.5 s) and fast (individuals with a TD ≤1.5 s). Change values (ΔMCAv, ΔCPP, Δ CVCi) are the differences from baseline and the last 30 s averaged of 50 W cycling for each variable. ΔMCAv_N_ and ΔCPP_N_ are the change from baseline to the nadir. ΔCPP_@MCvN_ and ΔCVCi_@MCAvN_ are the change from baseline to the value recorded when MCAv_N_ occurred. Autoregulatory Compensation index (ARCi), a calculated measure for autoregulation during MCAv_N_. Data are represented as mean (SD). *Denotes significant difference from slow, *p* < 0.05.

### 3.6 Sex had no effect on MCAv kinetics

Using the smallest effect size of interest (see Methods), we determined that the TD was equivalent (*t*(21) = −2.783, *p* = 0.006), tau was equivalent (*t*(21) = −2.931, *p* = 0.004), and MRT was equivalent (*t*(21) = −2.723, *p* = 0.006) between males and females. Additionally, MCAv_N_ (*t*(21) = 2.981, *p* = 0.004) and CPP_N_ (*t*(21) = −2.275, *p* = 0.017) were both equivalent as well. Therefore, we elected to not pursue any additional testing regarding possible sex differences.

## 4 DISCUSSION

The principal finding of the current study is that during a rest‐to‐exercise transition, MCAv briefly drops, which alters the dynamic variables described by a monoexponential model. Our data showed that MCAv_N_ was negatively correlated with TD (*r* = −0.560) and that MCAv_N_ was well predicted by CPP_N@MCAvN_ only ($R_{a}^{2}$ = 0.25, S = 4.74) compared to the full model ($R_{a}^{2}$ = 0.36, *S* = 4.97). This suggests that CPP has a direct impact on MCAv_N,_ which can alter the TD reported within a monoexponential model. During this time, CVCi rapidly increases, reaching its peak after TD (46.9 s vs. 20.2 s), and then settles near baseline for the remainder of the bout. These data indicate that large cerebral vascular responses are occurring shortly after the start of exercise, and the use of a monoexponential model masks this and reports it as a TD.

### 4.1 Monoexponential MCAv responses

In agreement with previous studies, we report a monoexponential model displayed a good fit to MCAv rest‐to‐exercise transition data ($R_{a}^{2}$ = 0.61) which is similar to Billinger et al. (2017) who reported an *R* ^2^ = 0.82. The difference can likely be attributed to a few methodological differences between our study and Billinger's. We chose to report $R_{a}^{2}$ instead of *R* ^2^ to account for bias and improve comparison (Akossou & Palm, 2013), and we used a 0.2 Hz lowpass filter for our data whereas Billinger et al. (2017) used a shape‐preserving, piecewise cubic interpolation. Billinger et al. also chose to increase subject's work over 30 s to achieve the desired work rate on a recumbent stepper, whereas subjects in our study began peddling on a recumbent cycle ergometer from rest immediately at 50 W. Together, these data indicate that a monoexponential model is a good tool for MCAv transition analysis. However, there is the unresolved issue of an extended TD. Previous studies have reported a TD of 30–50 s when using moderate‐to‐high intensity recumbent stepping (Billinger et al., 2017; Ward et al., 2018) and upright cycling (Weston et al., 2022) in young healthy adults. We show TD was negatively correlated with MCAv_N_ (*r* = −0.560), indicating a longer TD was related to a larger drop in MCAv (i.e., a more negative MCAv_N_). Therefore, to explain the extended TD, we need to explain why MCAv reaches a nadir at the onset of exercise.

### 4.2 Fluctuations in MCAv

We are not the first to report on the initial fluctuations in MCAv at the start of exercise. Weston et al. (2022) seem to have been the first group to shed light on this occurrence. During their monoexponential analysis, they chose to only model the exponential increase due to the oscillations occurring at the start of exercise. Although they did not directly attempt to describe this initial phase, they pointed at pressure and EtCO_2_ as primary factors in MCAv oscillations given the cerebrovascular responses to changes in EtCO_2_ (Hoiland et al., 2019) and pressure (Favre & Serrador, 2019; Kay & Rickards, 2016; Labrecque et al., 2019; Ogoh et al., 2022). We attempted to answer this question within the constraints of the study protocol. To do this, we applied a multiple regression to MCAv_N_ (Table 4). Within this model, EtCO_2@MCAvN_ was used and was not a significant predictor of MCAv_N_. In human cerebral vessels, CO_2_ is a potent vasodilator (Smith & Ainslie, 2017; Willie et al., 2014). However, EtCO_2_ values only increased by 5.73 ± 2.64 ΔmmHg during 50 W recumbent cycling (Figure 1d). It is unlikely that MCA diameter changed much, if any, at these low values (Verbree et al., 2014). Additionally, given the exercise‐induced hypotension (Figure 1b), cerebrovascular reactivity may be altered and therefore complicates the relationship (Willie et al., 2014). We cannot expand on this topic further; however, more testing to specifically answer this relationship is an interesting topic to broach.

The only significant predictor of MCAv_N_, that did not contain MCAv in its mathematical definition, was CPP_@MCAvN_ (Table 4). Therefore, we performed a linear regression with CPP as the only predictor (Figure 2a). These regressions had a similar fit (AIC = 140.4 vs. 140.7, Full model vs. CPP only) and were able to explain a similar amount of variance in MCAv_N_ ($R_{a}^{2}$ = 0.36 vs. 0.25, Full model vs. CPP only). Pressure displaying the strongest relationship with MCAv at exercise onset is similar to previous research (Ogoh et al., 2022; Saito et al., 2022). Although historically Q is thought to be one of the strongest determinates of CBF (Ide et al., 1998; Meng et al., 2015; Ogoh et al., 2005), Saito et al. (2022) found this is only true when analyzing the overall change from baseline to steady state. When analyzing the initial change from baseline to the first 40 s of 20 W cycling exercise, MAP was the strongest correlate (Saito et al., 2022). Additionally, Deegan et al. (2010) found that in the presence of dynamic CPP changes, Q was not a major contributor to changes in MCAv. Taken together, with the present data, we can conclude that as pressure drops at the start of exercise, MCAv is brought down with it. This drop in MCAv is not described by the monoexponential model, but instead attributes it to a TD.

### 4.3 Hypotensive response to exercise

The exercise‐onset hypotension we observe is not novel. A seminal study by Wieling et al. (1996) described a large drop in pressure 12 s after upright (−21 mmHg) and supine (−12 mmHg) 50 W cycling (Wieling et al., 1996). Similarly, we observed that CPP_N_ (−13.5 mmHg) occurred 10.3 s after 50 W recumbent cycling onset. Changes in TPR at the onset of exercise are likely the main contributor. Saito et al. (2022) noted systemic vascular resistance reduced towards a nadir at the start of 20 W upright cycling exercise. Recent work has suggested that skeletal muscle activation reduces TPR through rapid vasodilation (Michelini et al., 2015; Tschakovsky & Sheriff, 2004) and through loading of the atrial baroreflex from the skeletal muscle pump (Barbosa et al., 2015; Katayama et al., 2020; Tschakovsky & Sheriff, 2004). The magnitude of the drop in pressure is likely intensity‐driven. Although it does not appear to have been directly analyzed, Figure 4 in Barbosa et al. (2016) demonstrates an increased variability and lower nadir in MAP following moderate‐intensity exercise compared to low‐intensity exercise.

The dramatic and sudden drop of MAP at the start of exercise has a direct effect on MCAv. By activating the metaboreflex through occlusion of the forearm following handgrip exercise, Ogoh et al. (2022) attenuated the drop in MAP and MCAv following 20 W cycling exercise onset. This indicates that the atrial baroreflex alters sympathetic outflow. In their study, they found the relationship between the percentage drop in MCAv and the percentage drop in MAP to have an *R* ^2^ = 0.28, similar to the current study ($R_{a}^{2}$ = 0.31; Figure 2b). These combined actions (pressure and MCAv dropping) would, therefore, increase the calculated TD, indicating that the calculated TD is intensity‐driven. However, not all studies agree that exercise intensity alters TD. Recent evidence directly comparing moderate (80 W) to heavy intensity (159 W) MCAv kinetic data showed that TD values were similar (29.3 vs. 29.1 s) in young healthy adults (Weston et al., 2022) and in middle‐aged adults using similar workloads (91 W vs. 115 W) the recorded TDs were not different (43.5 vs 43.8 s) (Witte et al., 2019).

### 4.4 Cerebrovascular response

During the exercise‐onset hypotensive response, cerebral autoregulation would likely cause the cerebrovasculature to vasodilate to maintain flow (Koller & Toth, 2012; Payne, 2016). In the present study, we demonstrate this with CVCi (Figure 1c), an estimate of cerebral vasodilation and vasoconstriction (Burley et al., 2021; Claassen et al., 2007). At exercise onset, CVCi rapidly increases and reaches a peak, after MCAv_N_ (46.9 vs. 16.5 s), then declines and remains near baseline throughout the remainder of the exercise bout (Figure 1c). Not only does this highlight the necessity to focus on more than the steady state, but it also demonstrates the principal motivation for this study. Attributing the initial oscillations to a TD, as is the case with the current monoexponential model, masks the largest and most dynamic response of the cerebrovasculature that occurs during a rest‐to‐exercise transition. As MCAv and CPP move towards nadirs, this activates various vascular mechanisms that result in vasodilation to maintain flow (Koller & Toth, 2012). We attempted to quantify the ability of the cerebrovasculature to maintain MCAv during the drop in CPP, by calculating ARCi. Had CVCi not changed from baseline (CVCi_BL_), the MCAv would have dropped further (Chaudhry et al., 2023). ARCi quantifies how much autoregulation compensates for the drop in CPP to maintain MCAv. In the present study, we calculated ARCi to be 3.5%. This means that cerebral autoregulation was able to mitigate MCAv from dropping a further 3.5%. Although this does not seem very high, it is important to note that the cerebrovasculature maintains a more passive autoregulation in response to sudden drops in pressure as compared to increases in pressure (Claassen et al., 2021; Tan, 2012).

### 4.5 Fast vs. slow responders

Our data suggest that the subjects may be separated into two groups, “fast” and “slow,” when using a monoexponential model to fit the data. Several subjects displayed a rapid increase in MCAv (Figure 3a) at exercise onset (fast, *N* = 8) and others a more “typical” response (slow, *N* = 15). These subjects were of similar fitness levels and had similar resting MCAv values. The main difference demographically was that the slow group was more physically active (5549 vs. 2714 MET‐min/wk). Their TD values (30.2 vs. 1.5 s, slow vs. fast) and MCAv_N_ (−7.3 vs. 2.0 Δcm/s) were different between the groups, aiding in the argument that TD is being driven by large fluctuations in MCAv. It is important to note that the recorded MCAv_N_ for the fast group is a positive number (2.0 ± 2.6 Δcm/s). This is because for most fast responders, MCAv increases nearly instantly, and the “lowest value” being recorded for these individuals is during the exponential increase. We continue to use the term “nadir” for this group primarily for comparison purposes.

In Figure 3a, a “spike” can be observed in both groups, occurring just after MCAv_N_ for the slow. This is likely a result of the well documented Q “spike” or “overshoot” that occurs at the start of submaximal exercise (Saito et al., 2022; Wieling et al., 1996). As large‐muscle mass, upright exercise starts, Q increases reaching its peak at around 20 s, then reduces and is adjusted until it settles into a steady‐state (Saito et al., 2022; Wieling et al., 1996). At the start of exercise, the skeletal muscle pump loads the atrial baroreflex, resulting in reduced sympathetic function (Katayama et al., 2014). Saito et al. (2022) speculated this may indirectly protect the cerebral circulation by dampening the effects of Q increasing rapidly at the start of exercise. They also note that individuals with altered sympathetic control could have “abnormal CBF responses to the onset of exercise.” Although CPP_N_ between the groups was not statistically different (−16.1 vs. −8.7 ΔmmHg, *p* = 0.106), they were not equivalent (*t*(21) = 1.326, *p* = 0.0995), suggesting had there been a larger sample of fast subjects we may have found CPP_N_ to be different between the groups. If CPP_N_ was greater in the fast group, it also appears they had a greater “spike” in MCAv at the start of exercise (Figure 3a). These groups differed in physical activity and overall CPP response (8.6 vs. 17.3 ΔmmHg). Elevated exercising pressure has been linked to various cardiovascular disease risks (Dipla et al., 2017). We must be cautious in drawing conclusions with these data as it was purely exploratory and underpowered. However, the MCAv response in the fast group seems to be novel. The possibility that several young adults that are healthy, free of any cardiometabolic disease had what appears to be an abnormal MCAv response to exercise highlights the importance of examining the initial fluctuations in MCAv.

## 5 EXPERIMENTAL CONSIDERATIONS

We have attempted to limit confounding variables to the best of our ability; however, we acknowledge that there are several methodological considerations for this study. First, all female participants in our study were observed during the early follicular phase of the menstrual cycle to reduce possible cardiovascular hormonal influences (D'Urzo et al., 2018; Kellawan et al., 2015; Krejza et al., 2003; Limberg et al., 2010; Parker et al., 2007) even though there is a lack of evidence that menstrual cycle phase effects cerebrovascular control (Favre & Serrador, 2019; Krejza et al., 2003). We opted for a cautious approach to reduce any possibility that menstrual cycle phase would complicate data interpretation.

Secondly, TCD measures blood flow velocity through the MCA and does not account for vessel diameter. Therefore, our study did not directly measure volumetric blood flow. The cross‐sectional area (CSA) of conduit cerebral vessels will change in response to hand‐grip exercise and changes in arterial gases (Kellawan et al., 2016, 2017;Verbree et al., 2014, 2017). However, whether or not the MCA CSA changes during large muscle mass exercise remain unclear as cerebrovascular mechanisms responding to changes in perfusion pressure and arterial gases would be in opposition to each other (Smith & Ainslie, 2017). However, the values of EtCO_2_ observed in this study and the light exercise intensity would result in negligible changes to MCA CSA (Verbree et al., 2014, 2017). Conversely, more advanced imaging techniques such as MRI would not provide a temporal resolution required to answer the research questions addressed in this investigation (Wåhlin et al., 2013). Therefore, despite these limitations, we would argue that TCD was the superior instrument providing a surrogate for MCA blood flow during exercise.

Additionally, the data analyzed and expressed here are based off one exercise bout. Previous studies analyzing MCAv with a monoexponential model typically have used three repeated bouts in order to reduce signal‐to‐noise ratio (Billinger et al., 2017; Weston et al., 2022). This is not universal, and monoexponential modeling of a single‐bout of MCAv rest‐to‐exercise transition data has been done previously (Ward et al., 2018; Witte et al., 2019). The concept of averaging multiple bouts is done specifically to account for breath‐by‐breath variations (Poole & Jones, 2013; Whipp et al., 1982). It is unclear if the same technique is required of non‐ventilatory data. However, we do recognize that averaging of several bouts would reduce variability and likely increase understanding.

## 6 CONCLUSION

This study found that TD is directly correlated with MCAv_N_ and fluctuations of MCAv at the start of exercise are likely a result of an exercise‐onset hypotension reducing CPP. Cerebral autoregulation suggests that the cerebral vasculature responds to MCAv and CPP declining towards a nadir by rapidly dilating to maintain a consistent CBF. We estimate this response using CVCi, and show at the beginning of exercise, CVCi increases (suggesting vasodilation) towards CVCI_M_, then settles back towards baseline where it remains for the duration of the exercise bout. Characterizing this initial phase as a delay masks this large vascular activity. The ability of cerebral vessels to autoregulate and maintain blood flow has been well established in the development of neurodegenerative diseases (Claassen et al., 2021). Therefore, these initial oscillations should be more closely investigated instead of being attributed to a TD (Billinger et al., 2017; Weston et al., 2022; Witte et al., 2019). We suggest that analysis of MCAv dynamics should be accompanied by an analysis of CPP to better understand the myriad of physiological changes occurring. Interpretation of MCAv kinetic variables without an understanding of CPP is difficult. For instance, TD appears to be slightly altered following a stroke compared to healthy adults (Billinger et al., 2021; Ward et al., 2018) and quicker following kidney transplants (Ward et al., 2022). The utility of a monoexponential model to characterize the transition of MCAv from rest‐to‐exercise is apparent. Such a model may help determine mechanistic contributions to CBF and aid earlier detection of maladaptive responses, leading to earlier treatment. However, before conclusions can be drawn, a better understanding of what these variables are telling us is warranted.

## CONFLICT OF INTEREST STATEMENT

The authors declare no conflicts of interest.

## Supporting information


Figure S1.
