# Supplementary figures and images for: Cerebral blood flow dynamics: Is there more to the story at exercise onset?
Source: Physiol Rep. 2023 Jun 7;11(11):e15735. doi: 10.14814/phy2.15735 (PMC10247864; doi:10.14814/phy2.15735)

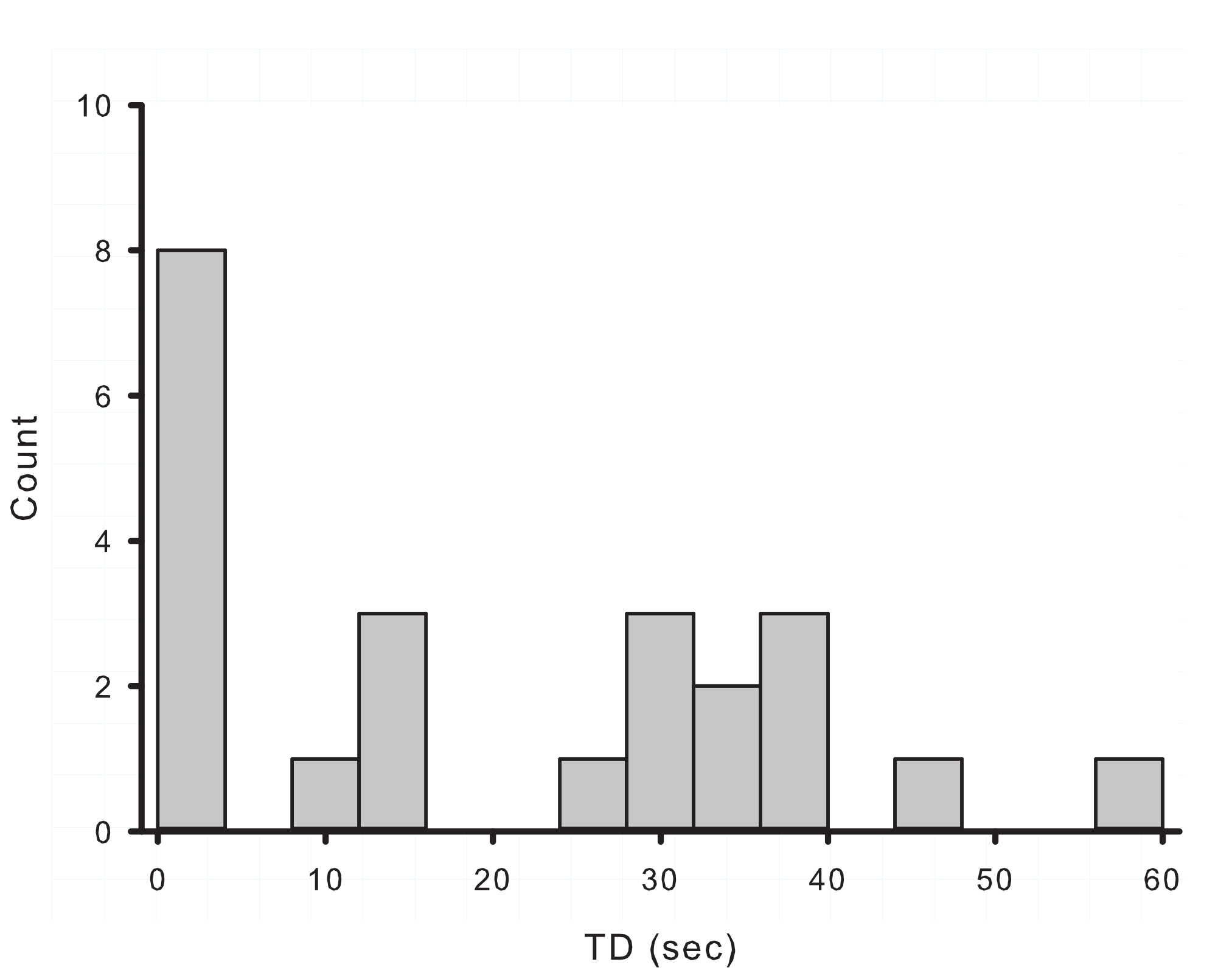

Supplement: Supplementary file 1 — Figure S1. [file PHY2-11-e15735-s001.tiff]
